# Dynamic Global and Local Enhancement of Theta‐Band Functional Networks as Neural Markers of Spinal Cord Stimulation Efficacy in Disorders of Consciousness

**DOI:** 10.1002/cns.71128

**Published:** 2026-08-30

**Authors:** Yinuo Liu, Qianqian Ge, Jiewei Lu, Yaqian Li, Jianda Han, Jianghong He, Ningbo Yu

**Affiliations:** ^1^ College of Artificial Intelligence Nankai University Tianjin China; ^2^ Institute of Intelligence Technology and Robotic Systems Shenzhen Research Institute of Nankai University Shenzhen Guangdong China; ^3^ Engineering Research Center of Trusted Behavior Intelligence, Ministry of Education Nankai University Tianjin China; ^4^ Department of Neurosurgery Beijing Anzhen Hospital, Capital Medical University Beijing China; ^5^ Department of Neurosurgery Beijing Tiantan Hospital, Capital Medical University Beijing China

**Keywords:** disorders of consciousness, dynamic functional connectivity, electroencephalography (EEG), spinal cord stimulation

## Abstract

**Background:**

Spinal cord stimulation (SCS) has emerged as a promising intervention for patients with disorders of consciousness (DoC). However, clinical optimization is hindered by a lack of interpretable prognostic biomarkers, and the neural mechanisms associated with recovery after SCS remain largely elusive.

**Methods:**

Nineteen DoC patients undergoing 70 Hz SCS were enrolled and categorized into effective (*n* = 9) and ineffective (*n* = 10) groups based on longitudinal clinical improvements. Resting‐state electroencephalography (EEG) was recorded across baseline, intra‐stimulation, and poststimulation phases. To investigate neural dynamics, theta‐band dynamic functional connectivity (dFC) was analyzed using the weighted phase lag index (wPLI) combined with a sliding window approach. Global and local dFC metrics, alongside region‐specific connectivity strengths, were quantified to characterize the global–local collaborative mechanisms induced by SCS.

**Results:**

Compared with the ineffective group, the effective group demonstrated enhanced global integration and decoupled regional segregation under SCS, evidenced by synchronized reduction in global average path length and suppression in local clustering (*p*_TPL_ = 0.0090; *p*_TLE_ = 0.0090; *p*_TCC_ = 0.0378). Regionally, increased functional connectivity was primarily observed in the right temporal cortex (*p* = 0.0054), whereas decreased connectivity was identified in the left occipital region (*p* = 0.0218). Furthermore, dynamic EEG features were significantly correlated with 3‐month CRS‐R improvement scores (*p* < 0.05).

**Conclusion:**

This dynamic global and local enhancement in the theta‐band dFC serves as an interpretable neural marker for predicting SCS efficacy. These findings offer novel insights into the system‐level collaborative mechanisms essential for consciousness restoration.

**Trial Registration:**

Chinese Clinical Trial Registry: ChiCTR2200061278

## Graphical abstract

To address the critical challenge of lacking objective prognostic neural markers for evaluating long‐term efficacy of spinal cord stimulation (SCS) in disorders of consciousness (DoC), this study proposed an EEG‐based dynamic functional connectivity (dFC) framework, uncovering SCS‐induced transient neural reorganization through higher‐order feature extraction and region‐specific analysis. Experimental results from 19 DoC patients revealed that SCS responders exhibited a distinctive global–local collaborative mechanism, characterized by enhanced global information integration alongside decoupled local segregation. These findings provided novel insights into the SCS‐induced recovery mechanisms and theoretical support for the early identification of potential plasticity responders.

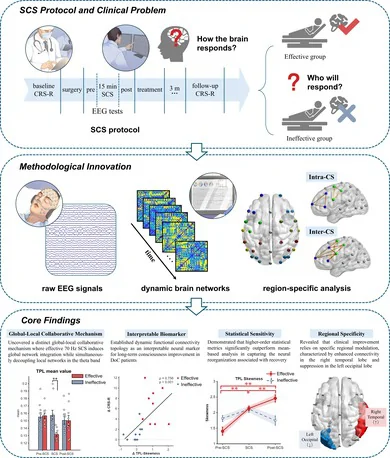

## 1 Introduction

Disorders of consciousness (DoC) refer to states of impaired consciousness resulting from severe brain injuries, primarily encompassing coma, vegetative state (VS), and minimally conscious state (MCS) [1]. These conditions pose significant challenges in clinical management and accurate prognosis [2, 3]. Spinal cord stimulation (SCS) stimulates the central nervous system via implanted electrodes, effectively promoting consciousness recovery [4]. It has been recognized as a promising treatment for DoC patients [5]. Piedade et al. [6] conducted a comprehensive analysis of 532 cases and found that SCS achieved an overall response rate of 47.9% in DoC patients. Qin et al. [7] identified two distinct mechanisms of neural plasticity in SCS, including compensatory activation and ipsilateral reorganization. However, the neural mechanisms underlying consciousness recovery after SCS remain incompletely understood [8], and its efficacy exhibits significant interindividual variability [9, 10]. Consequently, DoC prognosis prediction remains a core challenge in clinical neuroscience [11, 12].

The recovery of consciousness in DoC patients is intimately associated with dynamic integration and segregation within neural networks [13]. Current evidence indicates that their brain networks exhibit pathological activation patterns, characterized by disrupted temporal dynamics within the brain and abnormal persistence and transitions between different functional states [14, 15]. Bai et al. identified correlations between consciousness level and spontaneous transient brain states. Patients with different levels of consciousness exhibited significant differences in neural coordination patterns [14]. Zhuang et al. [15] demonstrated that single‐session SCS‐evoked EEG temporal dynamics were significantly associated with clinical improvement at 3‐month follow‐up, highlighting the potential of dynamic network metrics to reflect SCS delayed neuroplasticity. Despite these insights, the neural mechanism linking dynamic brain responses to stimulation with consciousness improvement remains unexplained [8, 16]. Traditional static features fail to capture the dynamic evolution, and single mean‐based analysis exhibits insufficient sensitivity in predicting prognosis [17, 18]. Dynamic functional connectivity (dFC) analysis of electroencephalography (EEG) can identify millisecond‐level transient network reorganization, reflecting dynamic coupling relationships between brain regions in critical frequency band [19, 20, 21]. This approach provides higher‐order markers for consciousness improvement, enabling more sensitive quantification of the intrinsic link between immediate brain responses and delayed neural effects.

The pathological basis of DoC is not homogeneous throughout the entire brain. Patients exhibit varying degrees of impairment in both global and local temporal stability [22], manifesting pronounced hemispheric regional specificity [23]. SCS exerts heterogeneous effects across different brain regions [24]. Consciousness recovery depends on not only global network integration but also functional remodeling of key brain regions and interhemispheric information transfer [25, 26, 27]. Luppi et al. [26] proposed a local orchestration of distributed functional patterns, wherein a few key local hubs coordinate large‐scale networks by regulating their own activity states and connections with others, thereby facilitating consciousness state transitions. Wu et al. [27] observed that DoC patients exhibited lower power ratios in primary regions compared to controls, along with significantly reduced directional connectivity between hemispheres. These asymmetric regulatory patterns might suggest functional divergences between bilateral cortical networks in SCS responsiveness. Single whole‐brain analysis risks obscuring critical region‐specific biomarkers and fails to explain local network reorganization [28]. Therefore, regional analyses enable precise localization of SCS regulatory effects on functional remodeling across regions, thereby revealing the global–local collaborative reorganization mechanism [29].

This study proposes an EEG‐based region‐specific dFC analysis method, extracting global and local network features as well as brain regional connectivity strength metrics. The aim is to investigate the intrinsic relationship between SCS‐induced dynamic brain functional reorganization patterns and consciousness improvement. Results from 19 DoC patients have indicated that effective SCS‐induced brain cortical responses are characterized by enhanced global integration and decoupled regional segregation, specifically manifested as increased connectivity in the right temporal lobe and suppressed in the left occipital lobe. By establishing interpretable neural markers of SCS efficacy, this study has provided a theoretical foundation for elucidating the recovery‐related neural mechanisms of SCS and facilitating individualized prognosis prediction for DoC.

## 2 Materials and Methods

### 2.1 Patients

Nineteen DoC patients were recruited from August 2021 to December 2023 at Beijing Tiantan Hospital, Capital Medical University. Before enrollment, the patients' immediate family members signed the written informed consent.

Inclusion criteria were as follows: (1) diagnosed as DoC; (2) age between 18 and 70 years regardless of sex; (3) onset of DoC more than 1 month before enrollment; (4) a stable consciousness level and conventional therapies for the recovery of consciousness were ineffective; (5) signed informed consent from family members to accept the SCS operation. Exclusion criteria were as follows: (1) DoC caused by neurodegenerative diseases and malignant brain tumor surgery; (2) coma caused by exacerbation of systemic diseases; (3) expected survival time less than 3 months; (4) epilepsy difficult to control without drugs; (5) spinal fracture or severe spinal stenosis. Major complications that could significantly affect recovery were either absent or well controlled prior to enrollment. Detailed baseline clinical characteristics are provided in Table S1. To reduce potential confounding effects, all patients were managed under a standardized treatment following the DoC treatment guideline.

The levels of consciousness in DoC patients were evaluated using the Coma Recovery Scale‐Revised (CRS‐R), with higher scores indicating better consciousness levels [30]. At least three preoperative assessments were conducted, and the highest score was taken as the baseline. Postoperative follow‐up assessments occurred 3 months after discharge, and the CRS‐R was obtained through inpatient, outpatient, and alternative evaluations at other medical institutions to observe the effect of SCS.

All patients were divided into effective and ineffective groups based on their consciousness level upon admission (baseline) and the recovery situation after SCS treatment (follow‐up assessment) as shown in Table 1. Specifically, the effective group was defined as patients who demonstrated an improvement of at least one level of consciousness state (e.g., from VS to MCS), whereas the ineffective group included patients with no change in consciousness level. No significant differences were observed in baseline scores, whereas the outcome in the effective group was significantly superior to the ineffective at 3‐month follow‐up assessment.

**TABLE 1:** Clinical characteristics of DoC patients enrolled.

Characteristics	Effective group	Ineffective group	*p*
Number	9	10	—
Age	37.8889 ± 17.8567	45.8000 ± 15.6191	0.4120
Sex	1 F, 8 M	5 F, 5 M	0.1409
CRS‐R (baseline)	8.3333 ± 3.3166	8.1000 ± 3.4785	0.3834
CRS‐R (follow‐up)	14.8889 ± 4.7022	9.3000 ± 4.2701	0.0057**

*Note:* The *p* values of age and CRS‐R were calculated by two‐sided nonparametric Wilcoxon rank‐sum test. The *p* value of sex was computed using two‐sided Fisher's exact test.

Abbreviation: CRS‐R, Coma Recovery Scale‐Revised.

** Significant differences (*p* < 0.01).

### 2.2 Surgical Procedure and Stimulation Protocol

**FIGURE 1:**
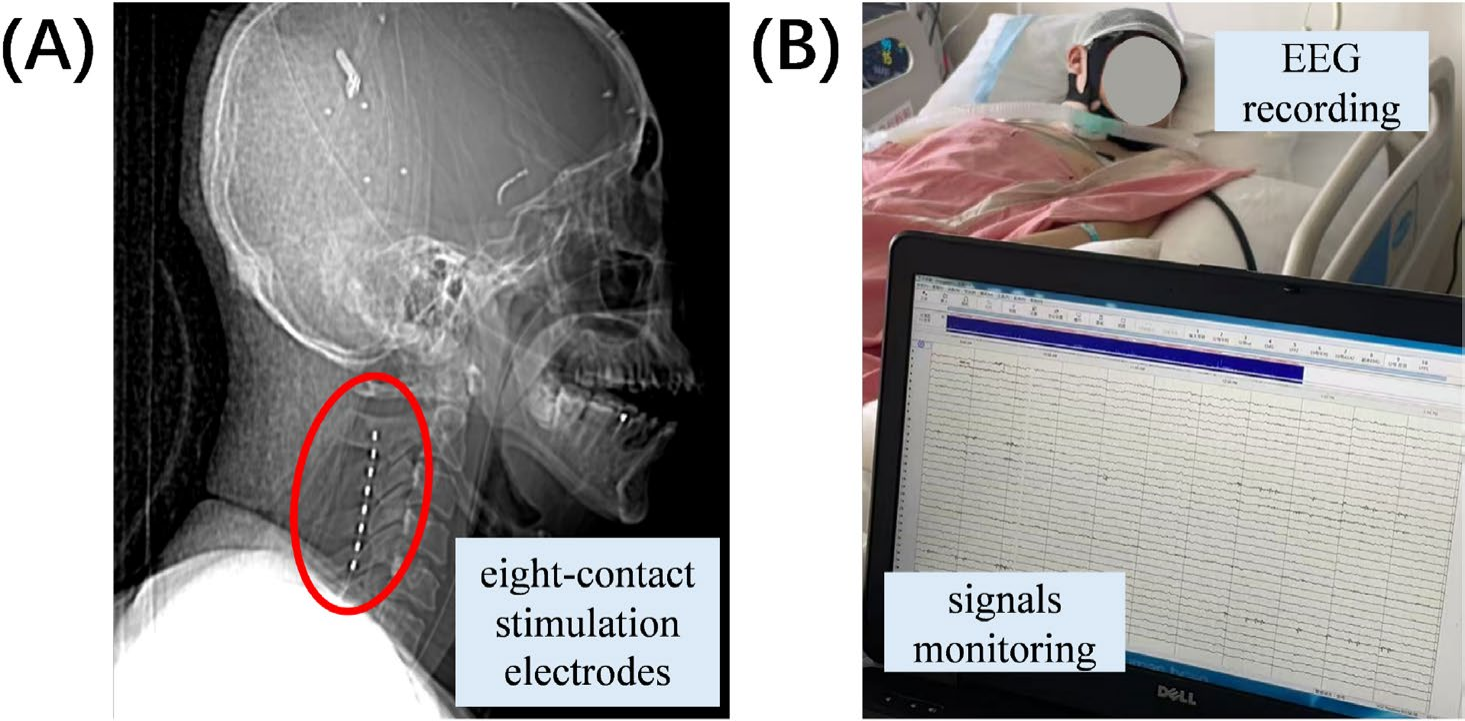
(A) Cervical CT 3D reconstruction after SCS implantation. The electrodes were advanced cranially along the midline into the epidural space and implanted at the C2 vertebrae level; (B) EEG tests were performed after surgery, including the baseline, a 15 min continuous SCS under EEG monitoring and the post‐SCS recording.

Following induction of general anesthesia, patients were positioned prone for percutaneous electrode insertion at the T7/8 intervertebral level (Figure 1A). Under X‐ray guidance, a single row of eight‐contact stimulation electrodes (model 3777; Medtronic) was advanced cranially along the dorsal midline into the epidural space at the C2 vertebral level. The electrode was subsequently interfaced with an external pulse generator and power source (model 37022; Medtronic). Cervical computed tomography was performed within 24 h after the operation to exclude complications. Upon completion of a 14 days stimulation period, electrodes were removed.

Stimulation parameters were set as follows: the uppermost three contacts configured in bipolar mode (0–, 1+, 2+), pulse width of 120 μs, frequency of 70 Hz, and intensity was set to a sub‐motor threshold without significant painful expression.

To assess neurophysiological responsivity, EEG tests were performed within 48 h after surgery across baseline, intra‐stimulation, and poststimulation phases (Figure 1B). Two electrophysiologists evaluated the alterations in EEG background activity independently to identify patients who were exhibiting positive neurophysiological responses for inclusion in the treatment protocol.

The therapeutic protocol spanned 14 days, delivered in cycles of 5 min on and 15 min off. To synchronize with the patients' sleep–wake rhythm, the stimulation was turned on at 8:00 a.m. and turned off at 8:00 p.m. every day.

### 2.3 EEG Recording and Preprocessing

EEG was recorded using a 32‐channel Nicolet amplifier (EEG V32; Natus Neurology) with Ag/AgCl electrodes arrayed in accordance with the international 10–10 system. The sampling frequency was 500 Hz. FCz and AFz respectively served as reference and ground electrodes. Electrode‐skin impedance was always maintained below 5 kΩ.

The raw EEG signals underwent offline preprocessing using the EEGLAB toolbox (version 2023.1) in MATLAB (version 2021a) [31] as follows: (1) application of a 1–40 Hz band‐pass filter and a 50 Hz notch filter to eliminate power line interference; (2) downsampling to 250 Hz; (3) removal of artifacts using Independent Component Analysis (ICA), in which stimulation‐related components are identified as those exhibiting large discrete temporal variance at 35 Hz/70 Hz and nonphysiological topographies; (4) use of re‐reference to eliminate bias introduced by the original reference; (5) segmentation of the data by defining the epoch as the entire stimulus period plus 300 s before and after the stimulus; (6) adjustment of the signal to a reference baseline level; (7) interpolation of bad channels; (8) inspection of the power spectral density (PSD) of the cleaned EEG signals to confirm that no stimulation‐related artifacts remained.

### 2.4 Dynamic Functional Connectivity Network Analysis

#### 2.4.1 Dynamic Brain Networks Construction

**FIGURE 2:**
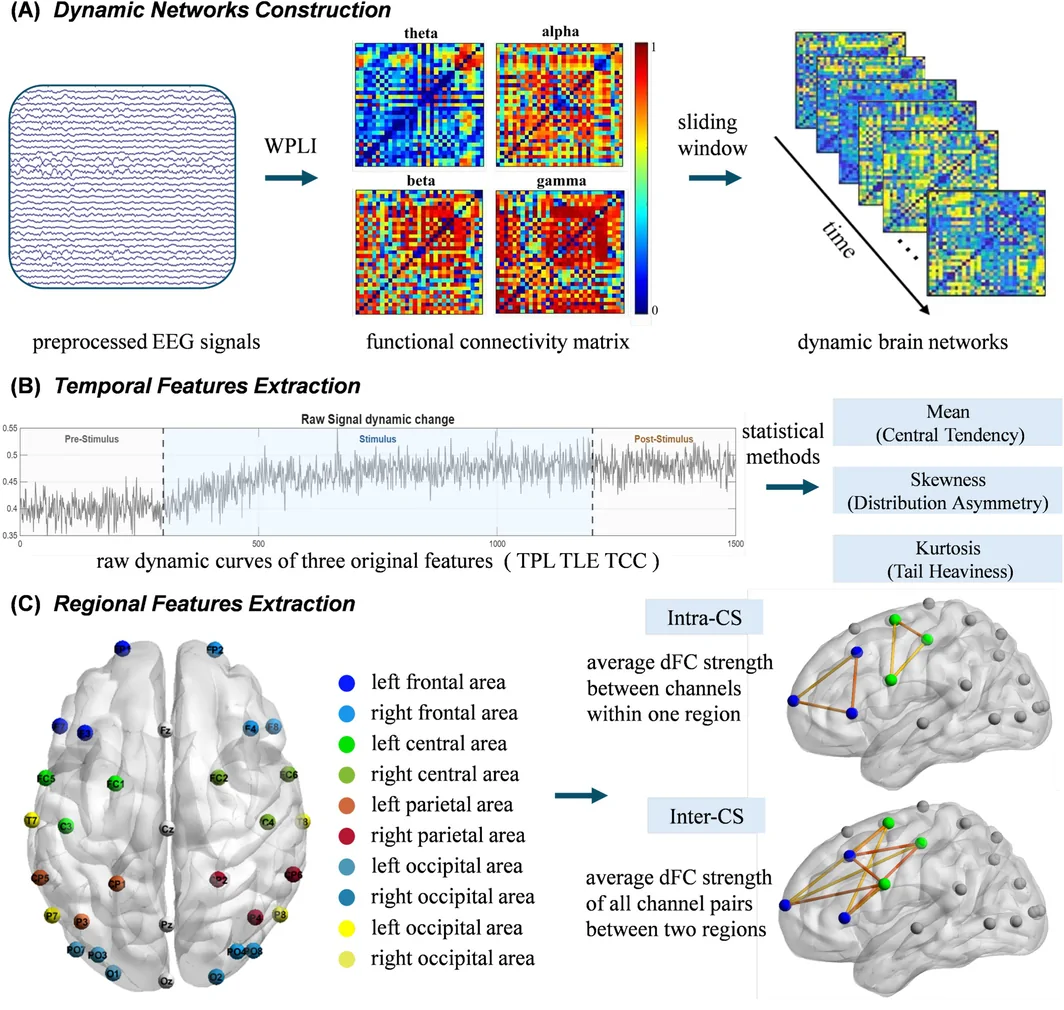
(A) The correlations between channels were calculated based on wPLI within each frequency band. Then, the dynamic brain networks were further constructed by using sliding windows. (B) Three fundamental metrics were calculated within each sliding window to form dynamic curves during the test period. Then, the dynamic distribution patterns of each metric were analyzed. (C) The raw signals were recorded using 32 electrodes arrayed in accordance with the international 10–10 system. All channels were further divided into 10 brain regions, with connectivity strength metrics calculated both within and between regions.

Dynamic brain networks were constructed by combining weighted phase lag index (wPLI) [32] and sliding window analysis, which involved the following steps (Figure 2A).

First, preprocessed EEG were divided into four frequency bands: theta (4–8 Hz), alpha (8–13 Hz), beta (13–30 Hz), and gamma (30–40 Hz) [33]. Neural oscillations at different frequencies correspond to distinct states of consciousness and brain functions. Constructing networks across different bands facilitates spectral analysis of brain activity changes.

Further, functional connectivity between channels was computed. wPLI quantified phase synchronization by assessing the weighted distribution of phase differences, thereby measuring connectivity strength between two EEG channels, which can be calculated as follows:

(1) $$\text{wPLI}=\frac{\left|\left\langle \left|\Delta \varphi_{\mathrm{xy}}\left(t\right)\right|\mathrm{sign}\left(\Delta \varphi_{\mathrm{xy}}\left(t\right)\right)\right\rangle \right|}{\left\langle \left|\Delta \varphi_{\mathrm{xy}}\left(t\right)\right|\right\rangle }$$

where *x* and *y* represented EEG signals from two electrodes within a given frequency band. wPLI values range from 0 to 1, with higher values indicating stronger synchronization between signals and greater coupling of neural oscillatory activity. Pairwise functional connectivity between channels ultimately presents as a two‐dimensional matrix.

Then, a dynamic functional connectivity matrix sequence was constructed based on sliding window analysis. The window length was set to 1 s [1, 34], with a step size of 1 s (nonoverlapping windows). This configuration ensured that each window contains at least 4–8 complete cycles of theta‐band oscillations, balancing spectral stability and temporal resolution. Nonoverlapping windows avoid artificial smoothing and provide unbiased distributional estimates for higher‐order statistical analysis [35].

For each temporal window, pairwise wPLI values between EEG channels were calculated to construct a dynamic functional connectivity matrix. In this matrix, each matrix element represented the functional coupling strength between a pair of EEG channels. Subsequently, the connectivity matrix was transformed into a weighted undirected graph by defining EEG channels as graph nodes and the corresponding wPLI values as weighted edges connecting node pairs. Through this node‐edge mapping process, the dynamic functional interactions among EEG channels were represented as time‐varying graph structures.

Finally, spatially and functionally related nodes collectively represented functional brain regions, while the interactions among these interconnected regions constituted the large‐scale dynamic brain network. Based on the resulting graph representations, graph‐theoretical network metrics were computed for each temporal window to characterize dynamic network organization over time. The complete dynamic functional connectivity sequence was ultimately represented as a three‐dimensional matrix with dimensions channels × channels × windows.

#### 2.4.2 Temporal Features Extraction

The dFC networks characterized neural temporal properties. Graph theory analysis was applied to extract metrics from the constructed networks. Both global and local properties were computed to quantify brain topological structure and dynamic changes. Three metrics were finally chosen: temporal average path length (TPL), temporal local efficiency (TLE), and temporal clustering coefficient (TCC).

TPL quantifies whole‐brain information integration efficiency by measuring the average shortest communication pathways between all node pairs [36]. The formula is:

(2) $$\mathrm{TPL}\left(t\right)=\frac{1}{N\left(N-1\right)}\sum_{i,j\in N,i\neq j}d_{\mathrm{ij}}\left(t\right)$$

where *N* is the total number of nodes and *d*_*ij*_ is the shortest path length between nodes *i* and *j*. TPL ranges from 0 to 1. Within brain networks, it reflects the ease or difficulty of integrating information across different regions, with shorter pathways indicating higher interregional communication efficiency.

TLE describes the efficiency of information transmission between neighboring nodes of a given node [36, 37]. The mean value of all nodes constitutes the network local efficiency. The calculation formula is:

(3) $$\mathrm{TLE}\left(t\right)=\frac{1}{N}\sum_{i\in N}\frac{\sum_{j,h\in N_{i},j\neq h}{\left[d_{\mathrm{ijh}}\left(t\right)\right]}^{-1}}{k_{i}\left(t\right)\left(k_{i}\left(t\right)-1\right)}$$

where *k*_*i*_ is the degree of node *i* and *d*_*ijh*_ indicates the shortest path length between node *j* and node *h* that exclusively includes node *i*. TLE ranges from 0 to 1. A larger value indicates higher information transmission efficiency around nodes and a higher degree of modularity across the entire network.

TLE concerns the efficiency of indirect connections, while TCC focuses on the presence of direct connections. TCC quantifies the clustering degree of nodes within dynamic networks, reflecting the compactness of connection structures at different time points [36, 38]. The calculation formula is:

(4) $$\mathrm{TCC}\left(t\right)=\frac{1}{N}\sum_{i\in N}\frac{2e_{i}\left(t\right)}{k_{i}\left(t\right)\left(k_{i}\left(t\right)-1\right)}$$

where *e*_*i*_ is the number of actual edges between the neighbors of node *i*. TCC ranges from 0 to 1. A higher value indicates a more compact local connection structure and more pronounced small‐world properties within the network.

In subsequent analyses, both mean values and statistical distribution features of these metrics were calculated (Figure 2B). The mean values characterized the overall network state across different stages, reflecting macro changes in information integration and separation. The calculation formula is:

(5) $$\mu =\frac{1}{M}\sum_{t=1}^{M}x_{t}$$

To capture the non‐Gaussian properties of network reorganization, the skewness and kurtosis of dynamic metrics were further extracted as distribution parameters. The calculation formulas are as follows:

(6) $$\text{Skew}=\frac{1}{M}\sum_{t=1}^{M}{\left(\frac{x_{t}-\mu }{\sigma }\right)}^{3}$$

(7) $$\text{Kurt}=\frac{1}{M}\sum_{t=1}^{M}{\left(\frac{x_{t}-\mu }{\sigma }\right)}^{4}$$

The dual‐layer approach enabled comprehensive evaluation of both persistent network reorganization and transient dynamic fluctuations.

#### 2.4.3 Regional Features Extraction

To further investigate the relationship between brain region‐specific connectivity and consciousness improvements, all 32 channels were divided into five primary regions of interest (ROI) as shown in Table 2 [39, 40, 41, 42, 43, 44, 45, 46]: frontal area, central area, parietal area, occipital area and temporal area. Each region was further subdivided into left and right brain areas (Figure 2C). The four midline electrodes were treated as independent midline nodes and excluded from the lateralized ROI‐based calculations.

**TABLE 2:** Brain regions of interest.

ROI	Channels	Functions
Left frontal	Fp1, F3, F7	Language production, cognitive control, executive functions [39]
Right frontal	Fp2, F4, F8	Spatial processing, attention orientation, negative emotion processing [40]
Left central	FC1, FC5, C3	Right motor execution, motor control and planning [41]
Right central	FC2, FC6, C4	Left motor execution, somatosensory anticipation and processing [41]
Left parietal	CP1, CP5, P3	Right spatial attention and orientation, goal‐directed selective attention [42]
Right parietal	CP2, CP6, P4	left spatial attention and orientation, stimulus‐driven attention [42]
Left occipital	PO3, PO7, O1	right visual processing, early visual perception, attention modulation [43]
Right occipital	PO4, PO8, O2	Left visual processing, self‐monitoring and awareness [44]
Left temporal	T7, P7	Language comprehension an memory [45]
Right temporal	T8, P8	Auditory processing, emotion processing, self‐awareness [45, 46]
Central nodes	Fz, Cz, Pz, Oz	—

Abbreviation: ROI, regions of interest.

Based on the constructed dynamic network sequences and brain region segmentation, Intra‐ROI Connectivity Strength (Intra‐CS) and Inter‐ROI Connectivity Strength (Inter‐CS) were extracted to quantify brain region‐specific effects [47].

Intra‐CS calculates the average connectivity strength between channels within a specific brain region, which can be defined as follows:

(8) $$\text{Intra}‐CS_{i}=\frac{1}{S}\sum_{m,n\in {\mathrm{ROI}}_{i}}{\text{WPLV}}_{m,n}$$

where *i* refers to the ROI number, *m* and *n* refer to two channels within the region. Intra‐CS ranges from 0 to 1, with higher values indicating greater information exchange intensity within that region.

Inter‐CS calculates the correlation between ROIs, which can be defined as follows:

(9) $$\text{Inter}‐{\mathrm{CS}}_{i,j}=\frac{1}{M\cdot N}\sum_{m\in {\mathrm{ROI}}_{i},n\in {\mathrm{ROI}}_{j}}{\text{WPLV}}_{m,n}$$

where *i* and *j* refer to the ROIs. Inter‐CS ranges from 0 to 1, with higher values indicating stronger information transmission between two brain regions.

### 2.5 Statistical Analysis

Normality of all features was assessed using the Shapiro–Wilk test (*α* = 0.05). For intergroup (independent‐sample) comparisons, features whose normality was rejected in either group were compared using two‐sided Wilcoxon rank‐sum tests; otherwise, independent‐sample *t*‐tests were used. For intragroup (paired‐sample) comparisons, paired *t*‐tests were uniformly applied. Multiplicity was controlled with the Benjamini–Hochberg false discovery rate (FDR) correction. For whole‐brain metrics, the correction was applied jointly across the three recording phases. For interregional connectivity analyses, the correction was applied across relevant regional connections within each recording phase. Finally, Spearman's rank correlation analysis was applied to examine the correlation between changes in brain function characteristics and CRS‐R scale scores changes, with correlation coefficients expressed as *R* values. The statistical significance threshold for all tests was set at *p* < 0.05.

## 3 Results

### 3.1 Whole‐Brain Network Dynamics: SCS Enhanced Global Integration and Local Segregation in Prognosis‐Positive Patients

Compared with the ineffective group, theta‐band dynamic global and local network metrics exhibited significant intergroup differences following SCS intervention (Table 3).

**TABLE 3:** Statistical analysis results for two groups of dynamic features.

Groups	Features	Distribution patterns	Pre‐SCS	SCS	Post‐SCS	*p* (pre‐scs)	*p* (pre‐post)	*p* (scs‐post)
Effective group	TPL	Mean	0.1568	0.1317	0.1501	0.0029**	0.3209	0.0550
		Skewness	1.4034	2.1355	2.4564	0.0016**	0.0009**	0.0016**
		Kurtosis	5.2246	9.7019	11.3425	0.0014**	0.0055**	0.1296
	TLE	Mean	0.4132	0.4099	0.4111	0.7331	0.7331	0.7331
		Skewness	0.2989	0.4364	0.4865	0.0121*	0.0061**	0.0989
		Kurtosis	2.6648	3.2739	3.5043	0.0000**	0.0000**	0.0007**
	TCC	Mean	0.4075	0.4053	0.4055	0.9552	0.9552	0.9552
		Skewness	0.3084	0.4311	0.4996	0.0189*	0.0035**	0.0453*
		Kurtosis	2.7358	3.1572	3.1895	0.0034**	0.0496*	0.8370
Ineffective group	TPL	Mean	0.1551	0.1572	0.1506	0.6646	0.5017	0.5017
		Skewness	1.8128	2.1099	1.7455	0.0677	0.5844	0.0677
		Kurtosis	7.8097	9.5838	7.3414	0.1560	0.4556	0.1560
	TLE	Mean	0.4269	0.4265	0.4291	0.9077	0.9077	0.9077
		Skewness	0.3889	0.4628	0.3241	0.2923	0.2923	0.2923
		Kurtosis	2.9424	3.1743	3.0065	0.3324	0.4953	0.4304
	TCC	Mean	0.4212	0.4178	0.4225	0.6677	0.8335	0.6677
		Skewness	0.3759	0.4719	0.3727	0.4684	0.9575	0.4684
		Kurtosis	2.9561	3.2080	2.9572	0.2112	0.9923	0.2401

Abbreviations: SCS, spinal cord stimulation; TCC, temporal clustering coefficient; TLE, temporal local efficiency; TPL, temporal average path length.

* Significant differences (*p* < 0.05).

** Significant differences (*p* < 0.01).

**FIGURE 3:**
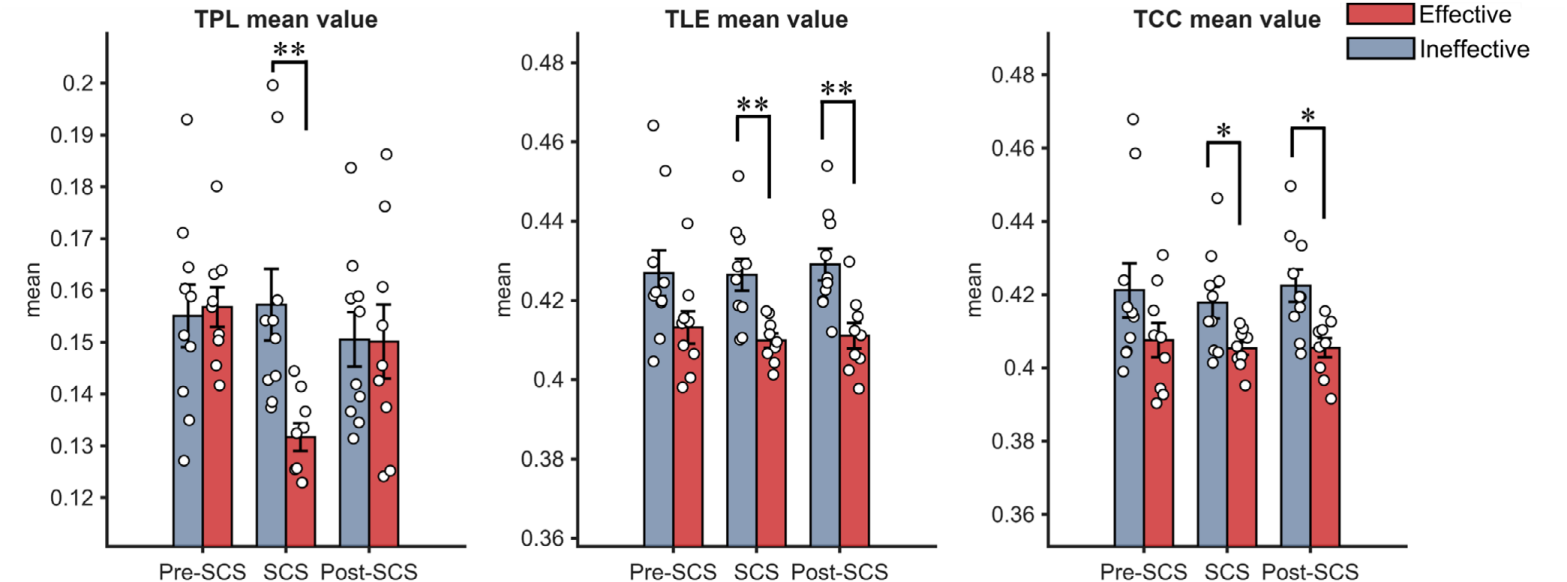
Intergroup differences in temporal metrics across stages showed global increase accompanied by local decrease in the effective group, while the ineffective group exhibited the opposite pattern (*p < 0.05, **p < 0.01).

Specifically, the mean value of theta‐band TPL decreased during stimulation in the effective group, whereas it increased in the ineffective group (Figure 3). During stimulation, TPL in the effective group was significantly lower than that in the ineffective group (*p* = 0.0090), indicating enhanced global network integration.

In contrast, the mean values of theta‐band TLE and TCC in the effective group were significantly lower than those in the ineffective group during stimulation (*p*_TLE_ = 0.0090; *p*_TCC_ = 0.0378). These intergroup differences remained observable during the poststimulation period (Figure 3).

Overall, the effective group exhibited reduced path length together with lower local segregation‐related metrics under SCS, whereas the ineffective group showed relatively elevated local network connectivity measures.

### 3.2 Distribution Statistical Patterns: Higher‐Order Statistics Outperformed Mean Values in Capturing Consciousness‐Related Reorganization

**FIGURE 4:**
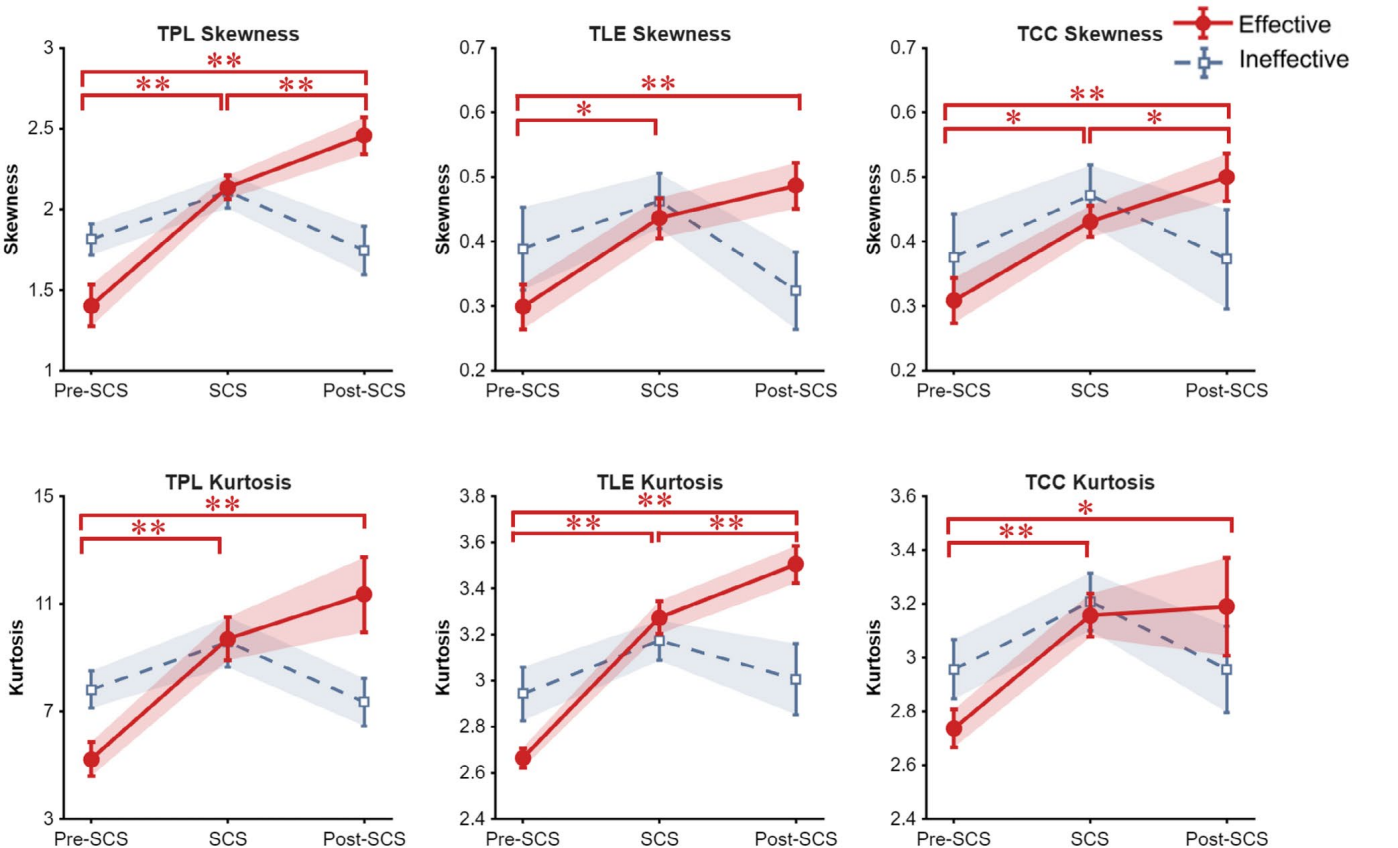
The effective group demonstrated significant changes in distribution patterns following stimulation, whereas the ineffective group did not exhibit this effect (*p < 0.05, **p < 0.01).

In the theta band, the mean values of local dynamic network metrics in the effective group remained relatively stable across different stages. In contrast, the skewness and kurtosis of these dynamic metrics increased significantly during stimulation relative to baseline. Most of these elevations persisted into the poststimulation phase (Figure 4).

By comparison, the ineffective group exhibited only transient increases in skewness and kurtosis during stimulation, and these changes returned toward baseline levels after stimulation cessation. No significant poststimulation changes were observed in the ineffective group.

Overall, higher‐order statistical metrics, including skewness and kurtosis, exhibited more pronounced temporal variations across stimulation stages than corresponding mean‐level metrics (Table 3).

### 3.3 Regional Specificity: Targeted Region Reorganization Distinguished Effective From Ineffective SCS Response

**FIGURE 5:**
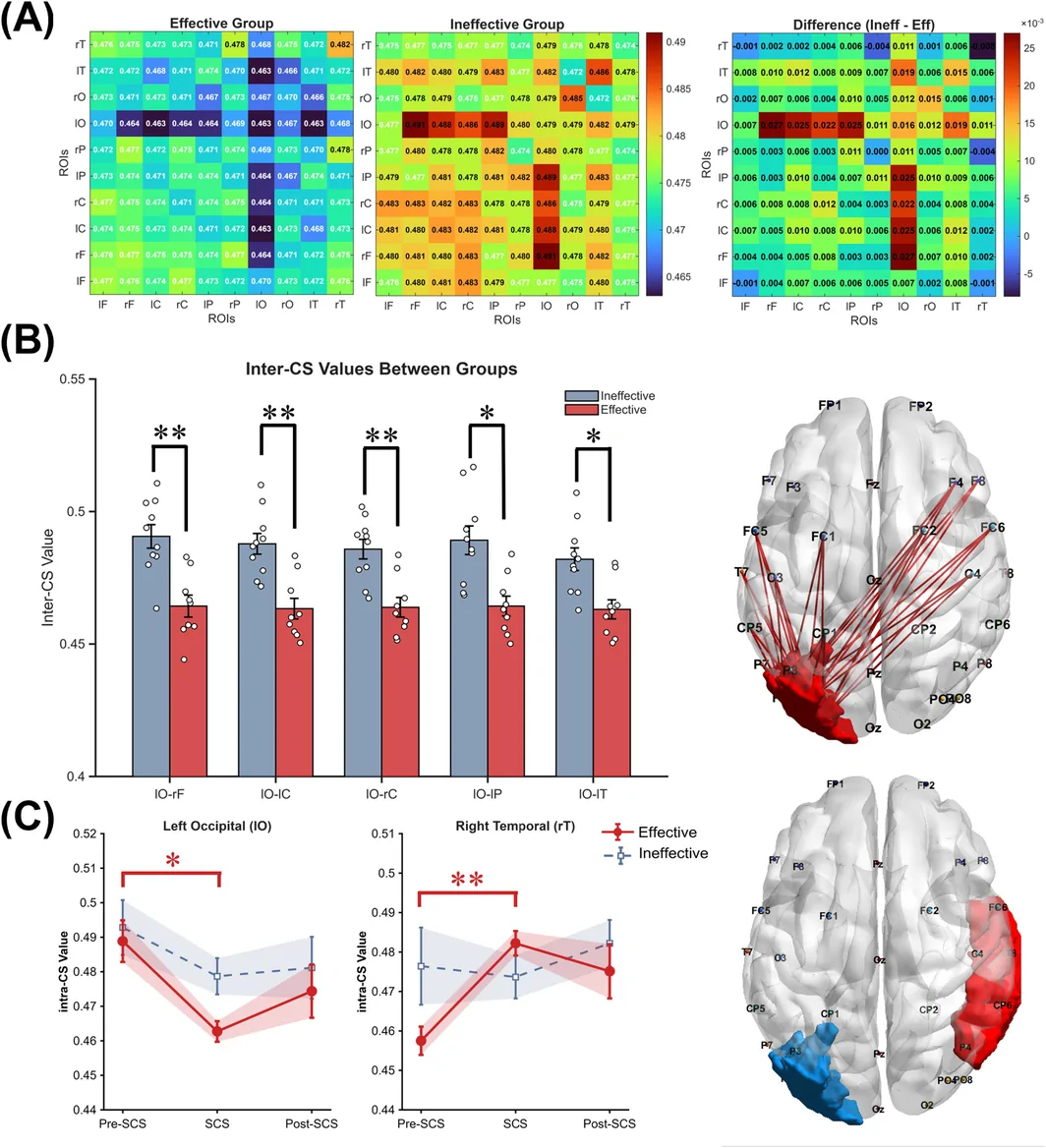
(A) Brain regional dFC strength and corresponding differences of two groups under SCS. (B) The ineffective group exhibited significantly higher inter‐CS metrics between the left occipital region and other brain regions than the effective group. (C) The effective group exhibited reduced intra‐CS metric in the left occipital lobe during stimulation, accompanied by enhanced connectivity strength in the right temporal lobe (*p < 0.05, **p < 0.01).

Intergroup comparisons revealed significant differences in theta‐band dFC between the effective and ineffective groups during stimulation (Figure 5A,B).

Specifically, compared with the effective group, the ineffective group exhibited significantly higher dFC between the left occipital region and the right frontal region (*p*_lO‐rF_ = 0.0041), bilateral central regions (*p*_lO‐lC_ = 0.0029; *p*_lO‐rC_ = 0.0045), the left parietal region (*p*_lO‐lP_ = 0.0101), and the left temporal region (*p*_lO‐lT_ = 0.0143). These inter‐CS differences remained observable during the poststimulation period.

Within‐group phase comparisons demonstrated distinct temporal dFC patterns between the two groups. The ineffective group exhibited widespread increases in dFC across multiple brain regions during stimulation. In contrast, the effective group showed regionally selective increases in dFC accompanied by reduced connectivity in several other regions.

Further regional analysis in the effective group demonstrated that the intra‐CS metric significantly decreased in the left occipital region (*p* = 0.0218) and significantly increased in the right temporal region (*p* = 0.0054) during SCS (Figure 5C).

### 3.4 Clinical Validation: EEG Changes Significantly Correlated With Consciousness Improvement

**FIGURE 6:**
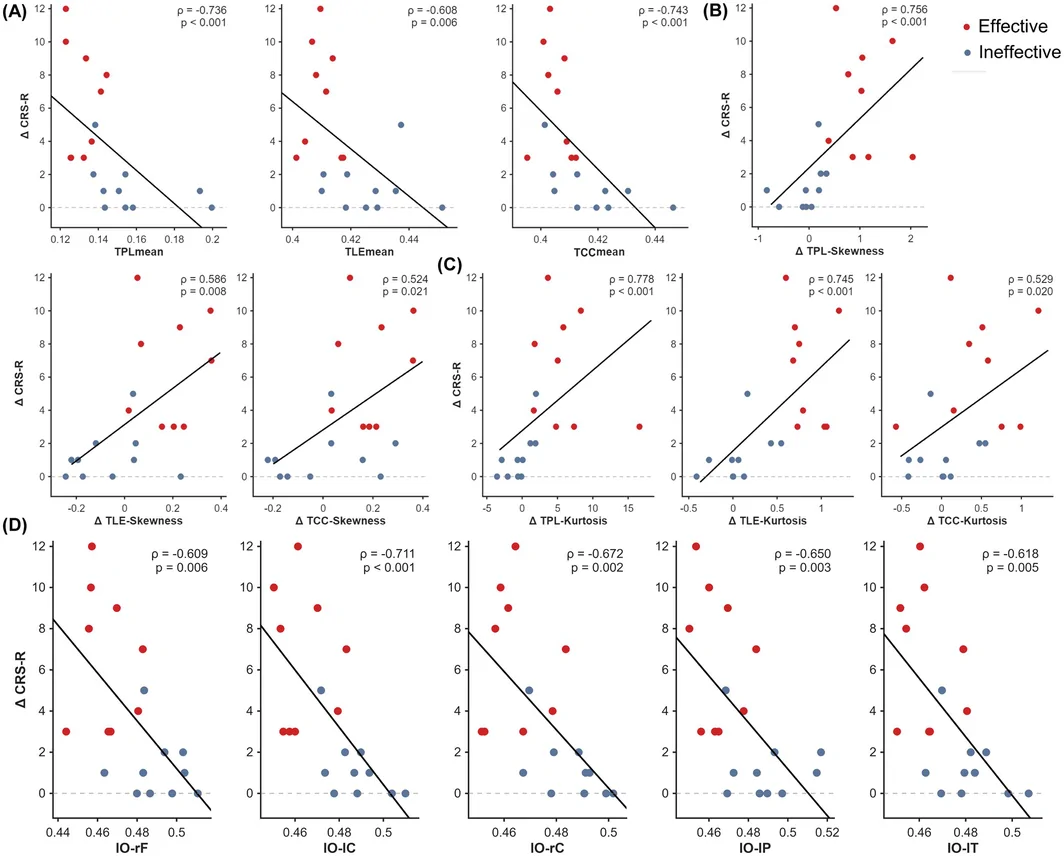
(A) Spearman correlation analysis was employed to examine the relationship between CRS‐R changes and dynamic features. The mean temporal metrics under SCS showed significant negative correlations with score changes, indicating that global enhancement and local suppression positively correlated with improved consciousness. (B) Changes in the kurtosis of temporal metrics showed significant positive correlations with score changes. (C) Changes in the skewness of temporal domain metrics also showed significant positive correlations with score changes; (D) Connectivity strength between the left occipital lobe and other brain regions showed significant negative correlations with score changes, meaning that patients with improved consciousness often exhibited reduced connectivity between this lobe and other regions.

Among whole‐brain network metrics, the dynamic mean values of the global metric (TPL) and local metrics (TLE and TCC) during SCS were significantly negatively correlated with ΔCRS‐R (Figure 6A).

In addition, higher‐order statistical features of these dynamic metrics, including skewness and kurtosis, showed significant positive correlations with ΔCRS‐R (Figure 6B,C). Specifically, increased skewness and kurtosis following SCS were associated with greater clinical improvement.

Among regional connectivity features, dFC strength between the left occipital region and multiple other brain regions exhibited significant negative correlations with ΔCRS‐R scores (Figure 6D). Reduced interregional connectivity involving the left occipital region was associated with greater improvement in consciousness level.

## 4 Discussion

### 4.1 Dynamic Functional Connectivity Analysis Captured Transient Neural Reorganization and Delayed Plasticity

This study confirmed that EEG‐based dFC analysis provided sensitive neural markers for predicting SCS treatment efficacy in DoC. Specifically, the mean dynamic features in theta band revealed significant differences in global and local connectivity patterns between the two groups. The effective group exhibited transient enhancement in whole‐brain information integration efficiency (TPL) and relative suppression of local connectivity (TLE, TCC) during SCS, whereas the ineffective group showed opposite patterns (Figure 3). These findings supported the theoretical framework that consciousness arises from dynamic interactions between brain regions rather than static anatomical connections [19, 48].

The enhanced information integration efficiency reflected strengthened overall information transmission capacity across distributed networks, laying the topological foundation for the global workspace required for consciousness recovery [29]. Meanwhile, the suppression of local connectivity suggested that SCS might promote a shift from rigid to flexible functional architectures by inhibiting pathologically hypersynchronized local clusters, which has also been observed in other brain diseases [49]. This dynamic global and local enhancement pattern aligned closely with subsequent regional results observing enhanced intra‐CS in the right temporal lobe and suppressed in the left occipital lobe (Figure 5C), collectively revealing that effective neuromodulation requires precise balancing of global efficiency and local specificity at the network level [50].

Changes in the statistical distribution of dynamic indicators demonstrated higher sensitivity (Figure 4) and showed significant correlation with clinical improvement at the 3‐month follow‐up (Figure 6B,C). Specifically, these higher‐order distributions of dynamic indicators (TPL, TLE, TCC) in the effective group exhibited significantly increased skewness and kurtosis under SCS and remained significantly higher than baseline in the poststimulation phase, whereas the ineffective group showed only transient fluctuations and finally reverted to baseline. These aligned with prior research emphasizing the relationship between non‐Gaussian metrics and different levels of consciousness [51]. The baseline‐relative elevation of kurtosis and skewness in the effective group indicated a more sharply distributed network state over time, suggesting their brain operated with a stronger tendency toward highly efficient connectivity patterns and potentially experienced more frequent transient states [15]. This effect may represent an early neurophysiological signature of plasticity, analogous to early‐phase long‐term potentiation (E‐LTP), whereby transient stimulation induces functional changes that persist beyond the stimulation period [52]. Classical studies suggest that E‐LTP is maintained through activity‐dependent strengthening of existing synaptic efficacy and serves as a critical substrate for subsequent long‐term network reorganization. Accordingly, the ability to maintain stimulation‐induced network configurations may indicate preserved neuroplastic capacity, potentially facilitating the delayed behavioral recovery observed after SCS treatment. The transient fluctuations and baseline regression observed in the ineffective group may suggest an inability of their brain networks to sustain such efficient dynamic reorganization. Excessive local connectivity further indicated the brain may be a rigid state of pathological instability [53]. Consequently, this research corroborated the findings of Della Bella et al. [54] that the emergence probability of high‐entropy connectivity patterns correlated significantly with the recovery of consciousness. Building upon the discovery of Zhuang et al. [15] that SCS‐induced temporal dynamics can predict delayed clinical improvement, we further indicated that morphological alterations in dynamic distributions could serve as key sensitive indicators for capturing conscious engagement and predicting prognosis, supporting the hypothesis that residual conscious activity might manifest as non‐Gaussian dynamic fluctuations rather than simple amplitude changes [53, 55].

The observed increases in skewness and kurtosis of theta‐band dynamic connectivity metrics can be interpreted within a neurophysiological framework. The concept of criticality posits that brain networks operate near a phase transition point, maximizing information capacity, dynamic range, and sensitivity to input. Near criticality, neural activity exhibits scale‐free correlations and non‐Gaussian fluctuations. In healthy conscious brains, neural activity operates near a critical point, balancing excitation and inhibition to maximize information integration and metastability [56, 57]. DoC are associated with a rigid, hypersynchronous state characterized by reduced metastability and a deviation from criticality [58].

Within this framework, effective SCS may facilitate a transition away from this pathological regime toward a more flexible and adaptive network state. This interpretation is supported by the emergence of asymmetric (increased skewness) and heavy‐tailed (increased kurtosis) distributions in transient connectivity strength. Specifically, increased positive skewness may reflect the occurrence of infrequent but high‐amplitude connectivity events, suggesting enhanced network reconfiguration capacity and transient departures from rigid synchronization patterns. Similarly, elevated kurtosis indicates a higher probability of extreme connectivity states, implying that the brain network intermittently accesses highly integrated configurations rather than remaining confined to persistently over‐synchronized activity patterns.

These higher‐order statistical changes may also reflect restoration of metastable neural dynamics. Metastability refers to the capacity of the brain to transiently visit multiple distinct functional configurations without becoming trapped in a single stable state [59]. From this perspective, skewness and kurtosis provide distribution‐level descriptors of the variability and flexibility of dynamic network organization. The post‐SCS elevation relative to baseline in these metrics observed in the effective group suggests an expanded repertoire of transient brain states and improved flexibility of global and local network reconfiguration. In contrast, the ineffective group showed absent or only transient changes in higher‐order statistics, indicating persistent impairment of metastable dynamics and limited recovery of adaptive network flexibility.

### 4.2 Brain Region‐Specific Responses Revealed the Global and Local Enhancement Mechanism of Recovery After SCS

This study revealed that the effective group demonstrated a specific pattern of intra‐CS enhancement in the right temporal lobe and suppression in the left occipital lobe, while the ineffective group exhibited widespread inter‐CS enhancement centered on the left occipital lobe. The lateralized regulatory patterns held significant theoretical implications for understanding the mechanisms associated with consciousness recovery after SCS.

The right temporal lobe played a central role in auditory processing, multimodal integration, emotional processing, and self‐referential consciousness [45, 46]. Previous studies have suggested that temporal cortical regions, particularly the temporal pole and medial temporal structures, constitute important components of several default mode network (DMN) [60, 61]. Preservation of DMN cohesive function has been correlated with the consciousness levels and DoC prognosis [62, 63]; therefore, the enhanced right temporal lobe connectivity observed in the present study may be consistent with the recruitment of network processes related to consciousness recovery. Enhanced intra‐CS within the right temporal lobe suggested that SCS might indirectly modulate cortical excitability through ascending activation systems [64]. This prioritized the reactivation of integrative hubs within the right hemisphere [46], facilitating the formation of a global workspace and aiding in the reconstruction of higher‐order integrative functions related to self‐awareness and environmental perception [65]. This aligned with the theory proposed by Luppi et al. that consciousness recovery involved local coordination of distributed functional patterns, where a few key hub regions supported consciousness emergence by orchestrating large‐scale network transitions [26].

The effective group exhibited reduced intra‐CS in the left occipital lobe, alongside significantly diminished connections to other brain regions compared to the ineffective group. This sensory information flow gating mechanism might reflect the visual cortex's decoupling from pathological synchronization patterns. Effective stimulation intervention prevented irrelevant or excessive low‐level sensory input from overwhelming already compromised higher‐level neural networks, thereby liberating resources for more efficient information integration [66]. This functional mechanism resonated with the structural findings of Sörös et al. [43], suggesting that left occipital cortical thickening in patients with attention deficits might represent a compensatory mechanism for impaired attentional network function. Conversely, the persistent hyperconnectivity between the left occipital lobe and other regions such as the frontotemporal cortex in the ineffective group might represent a pathologically inefficient compensatory hyper‐synchronization [67], which has also been observed by Pusil et al. [68] in mild cognitive impairment. The visual cortex remained locked in rigid and nonfunctional coupling, impeding flexible information routing. In severe brain injury, the primary sensory cortex often exhibited abnormal hyperconnectivity due to compensatory reorganization [69, 70, 71]. This hypersynchrony limited network differentiation and flexible restructuring, trapping networks in rigid states incapable of supporting the complex dynamics required for consciousness [72]. This results also provided a more detailed explanation for the higher mean values observed in the local features within the ineffective group.

By integrating dynamic whole‐brain and regional findings, this study proposed a pattern of enhanced global integration and decoupled regional segregation in DoC patients who were experiencing improved consciousness following SCS treatment. This aligned with the discovery of Plosnić et al. [73] that DoC patients were identified by diminished global information processing and increased local information processing. These findings suggested that optimal functional recovery typically required not only the activation of target networks but also the suppression of competing or maladaptive activities.

### 4.3 Further Understanding of the SCS Neural Modulation Mechanisms in Low Frequency

The findings of this study were primarily based on the theta band. While partial significances were also observed in other bands, the theta‐band metrics specificity proved particularly pronounced and extensive. SCS activated ascending sensory pathways projecting to the thalamus and reticular formation, which were the key structures for arousal regulation [7]. And theta oscillations played a pivotal role in top‐down regulation of thalamocortical integration and attention [74]. The observed theta‐band connectivity changes might reflect SCS‐induced enhancement of thalamocortical dialogue, thereby facilitating information integration across distributed cortical networks. This brain stability in the theta band has already been validated in existing research as a potential neural marker for predicting the recovery of consciousness [1].

Further, based on dynamic results, effective SCS arousal might depend on a refined collaborative mechanism. On the one hand, the stimulation moderately enhanced overall whole‐brain information transmission efficiency. On the other hand, it amplified functionality in critical areas while simultaneously suppressing nodes that might undermine network efficiency, thereby achieving optimal allocation of network resources [75]. By contrast, patients who were insensitive to SCS exhibited a deficiency in such precise regulation, being trapped in diffuse whole‐brain hyperconnectivity. Collectively, these findings could deepen the understanding of SCS neural modulation mechanisms across three levels as follows:

At the system level, enhanced global theta‐band integration supported the ascending activation hypothesis, where cervical SCS activated the spinothalamic tract and reticular formation, diffusely projecting to the cortex and amplifying consciousness‐related oscillatory coupling [76]. The baseline‐relative persistence of connectivity changes in the poststimulation phase suggested the induction of potentiation plasticity [77], rather than merely transient effects.

At the network level, the emergence of the right temporal lobe as a connective hub aligned with the global workspace theory of consciousness, which posited that conscious recovery required information broadcasting through the combined frontal–parietal and temporal cortices [78]. The SCS might facilitate this broadcasting by increasing the network centrality of the temporal lobe, enabling it to coordinate otherwise fragmented cortical modules.

At the dynamic level, altered non‐Gaussian distributions might reflect shifts in excitation–inhibition (E‐I) balance. Existing computational models suggested consciousness depended on critical E‐I dynamics [79, 80]. SCS‐induced theta modulation might drive cortical networks toward this critical state, enhancing dynamic repertoire and information capacity.

### 4.4 Clinical Significance and Prognostic Value

The correlation between dynamic EEG markers and 3‐month CRS‐R improvement suggested these indicators might serve as potential prognostic tools for screening and optimizing treatment in DoC. In clinical practice, this dFC approach could provide complementary functional insights for structural neuroimaging and behavioral assessments in detecting residual consciousness or predicting neuroplasticity potential.

Intraoperative theta‐band dFC features, particularly the increased skewness and kurtosis during trial stimulation, coupled with the E‐I pattern of specific lobes, might represent plasticity responders with a higher probability of benefit. These indicators could serve as early objective neural markers of SCS efficacy, supporting clinical decision‐making and the early identification of potential patients.

Regarding SCS treatment optimization, these findings provided theoretical support for personalized and targeted neuromodulation based on brain network feedback. EEG monitoring could assist in adjusting stimulation parameters to induce the specific network pattern of right temporal lobe enhancement along with left occipital lobe suppression. Furthermore, the right temporal lobe, as a potential key arousal node, could serve as a combined or alternative target for other neuromodulation techniques to explore multimodal therapies.

This study has several limitations. First, the sample size is relatively small (*n* = 19), which may limit the generalizability and robustness of the findings. In addition, the absence of a sham‐stimulation control group limits our ability to fully disentangle stimulation‐related effects from potential spontaneous recovery, particularly when interpreting long‐term clinical outcomes. Future work would expand the sample size through in‐depth collaboration and conduct larger‐scale studies with a control group to validate the reproducibility of these biomarkers. Additionally, subgroup analyses based on the baseline level of consciousness would be performed to evaluate the performance of the metrics across different patient subpopulations. Second, EEG recordings were conducted only during the testing phase and were not repeated at later time points. Future research will directly verify the existence of long‐term plasticity through follow‐up data collection. Finally, the study focused primarily on the theta band. Future work should systematically investigate cross‐frequency interactions and their role in consciousness recovery.

## 5 Conclusion

This study uncovered a distinct global–local collaborative mechanism underlying effective 70 Hz SCS in DoC, characterized by enhanced global network integration and simultaneous decoupling of local networks in theta‐band. These dFC topologies could serve as interpretable neural markers for consciousness improvement. Notably, higher‐order statistical metrics (skewness and kurtosis) significantly outperformed traditional mean‐based analysis in capturing the neural reorganization associated with consciousness restoration. Regionally, clinical improvement relied on a specific modulation pattern, characterized by increased connectivity in the right temporal lobe and suppressed connectivity in the left occipital cortex, contrasting with the diffuse hyperconnectivity observed in nonresponders. The baseline‐relative persistence of altered non‐Gaussian distributions in the poststimulation phase further indicated the plasticity effects of SCS on brain functional reorganization. Collectively, these findings provided potential objective neurophysiological evidence for understanding recovery‐related network mechanisms and identifying potential plasticity responders.

## Funding

This work was supported by the National Natural Science Foundation of China (U24A20284, 62503246, 62473214), the Tianjin Natural Science Foundation Project (25JCZDJC01020), the China Postdoctoral Science Foundation (2025M782920), and the Shenzhen Science and Technology Program (KQTD20210811090143060).

## Ethics Statement

This study was conducted in accordance with the Declaration of Helsinki established by the World Medical Association and approved by the ethics committee of Beijing Tiantan Hospital (No. KYSQ 2021‐396‐01).

## Conflicts of Interest

The authors declare no conflicts of interest.

## Supporting information


**Table S1:** Individual‐level clinical characteristics of DoC patients enrolled.

## Data Availability

The data that support the findings of this study are available on request from the corresponding author. The data are not publicly available due to privacy or ethical restrictions.
